# Cochlear Radiation Dose and Early Hearing Changes After Chemoradiotherapy for Head and Neck Cancer: A Single-Center Retrospective Cohort Study

**DOI:** 10.3390/jcm15207985

**Published:** 2026-10-15

**Authors:** Anda-Ioana Morgovan, Nicolae Constantin Balica, Cristina Mihaela Negru, Kristine Guran, Alexandru Orasan, Mihaela Andreea Banta, Crina Oana Pintea, Adrian Mihail Sitaru, Horatiu Eugen Stefanescu

**Affiliations:** 1 Department of ENT, “Victor Babes” University of Medicine and Pharmacy Timisoara, 300041 Timisoara, Romania; anda.morgovan@umft.ro; 2 Department of Otolaryngology, “Victor Babes” University of Medicine and Pharmacy Timisoara, 300041 Timisoara, Romania; balica@umft.ro (N.C.B.); guran.kristine@umft.ro (K.G.); alexandru.orasan@umft.ro (A.O.); andreea.banta@umft.ro (M.A.B.); crina.pintea@umft.ro (C.O.P.); adrian.sitaru@umft.ro (A.M.S.); stefanescu@umft.ro (H.E.S.)

**Keywords:** head and neck neoplasms, chemoradiotherapy, cisplatin, radiotherapy, hearing loss

## Abstract

**Background/Objectives:** Hearing impairment is a frequent but under-recognized sequela of (chemo)radiotherapy for head and neck cancer (HNC). We assessed the frequency of early post-treatment hearing changes and their association with cochlear radiation exposure and concurrent platinum-based chemotherapy. **Methods:** We conducted a single-center retrospective cohort study of 70 consecutive patients with HNC treated with radiotherapy between November 2024 and September 2025. Patients receiving concurrent chemoradiotherapy (CRT, *n* = 48) were compared with patients receiving radiotherapy alone (RT, *n* = 22). The primary outcome was clinically documented post-treatment hearing change. Cochlear maximum point doses (Dmax) were extracted from treatment plans. Analyses included Fisher exact and Mann–Whitney U tests, Spearman correlations, Firth penalized logistic regression, a Cochran–Armitage trend test, and restricted cubic splines. **Results:** Post-treatment hearing changes were documented in 38/70 patients (54.3%): 58.3% after CRT versus 45.5% after RT alone (odds ratio (OR) 1.68, 95% confidence interval (CI) 0.61–4.49, *p* = 0.439). Mean cochlear Dmax was higher in patients with hearing change (median 5.5 vs. 2.5 Gy, *p* = 0.045) and correlated with the outcome (ρ = 0.244, *p* = 0.043), with hearing-change rates of 34.8%, 65.2%, and 65.2% across cochlear-dose tertiles (trend *p* = 0.038). In Firth regression adjusted for age, pre-treatment hearing loss, and concurrent CRT, the association was attenuated and not statistically significant (adjusted OR 1.31 per 10 Gy, 95% CI 0.82–2.08, *p* = 0.253), and it was attenuated further after accounting for tumor site (OR 1.09 per 10 Gy, 95% CI 0.66–1.80, *p* = 0.735), with oropharyngeal primary the strongest correlate of hearing change (OR 4.02, 95% CI 1.28–12.6). Paired audiograms were available in only 10 patients, seven of whom showed audiometric deterioration; a conductive component could not be excluded in 14/38 events. No patient exceeded the QUANTEC mean cochlear-dose constraint of 45 Gy. **Conclusions:** Early, clinically apparent hearing changes affected more than half of this contemporary cohort. The univariable association with cochlear dose did not persist after adjustment, particularly for tumor site, and the available audiometric data do not allow hearing changes to be attributed specifically to cochlear injury. These findings underscore the need for systematic baseline and follow-up audiometry rather than demonstrating that further cochlear-dose reduction would improve hearing outcomes.

## 1. Introduction

Head and neck cancer (HNC) remains a major global health problem, with more than 900,000 new cases and over 450,000 deaths estimated annually worldwide, and squamous cell carcinoma accounting for the vast majority of cases [1]. The disease characteristically presents at a locally advanced stage, arises in anatomic subsites that are essential for breathing, speech, swallowing, and hearing, and predominantly affects patients with substantial tobacco and alcohol exposure and accumulating comorbidity [2]. Because both the tumor and its treatment involve densely innervated and functionally critical structures, survivorship in HNC is defined not only by oncologic control but also by the burden of long-lasting functional sequelae, among which auditory impairment occupies a prominent yet frequently neglected position.

Radiotherapy, either definitive or postoperative, is required in the majority of patients with locally advanced disease, and the addition of concurrent platinum-based chemotherapy improves overall survival, with an absolute benefit of approximately 6.5% at five years demonstrated by the Meta-Analysis of Chemotherapy in Head and Neck Cancer (MACH-NC) [3]. The updated MACH-NC analysis of 107 randomized trials and 19,805 patients confirmed that concomitant chemotherapy remains the most effective way of combining systemic treatment with irradiation, although the benefit attenuates with advancing age [4]. Landmark trials, such as the laryngeal-preservation study of concurrent cisplatin and radiotherapy, consolidated chemoradiotherapy (CRT) as the standard of care for organ-preserving treatment of advanced disease [5].

The oncologic gains of concurrent platinum come at the cost of additional toxicity, and the inner ear is among the most vulnerable targets. Cisplatin generates reactive oxygen species, activates apoptotic cascades in outer hair cells, the stria vascularis, and spiral ganglion neurons, and typically produces bilateral, progressive, and irreversible high-frequency sensorineural hearing loss [6]. The pharmacologic understanding of platinum-induced ototoxicity has advanced considerably, revealing dose-dependent cochlear injury with substantial inter-individual variability [7]. Importantly, cisplatin is retained within the cochlea indefinitely after systemic administration, providing a mechanistic explanation for cumulative and delayed auditory damage [8].

Radiation itself is independently ototoxic. The Quantitative Analyses of Normal Tissue Effects in the Clinic (QUANTEC) review of radiation-associated hearing loss recommended limiting the mean cochlear dose to ≤45 Gy, and more conservatively to ≤35 Gy when cochlear sparing is feasible, to reduce the risk of sensorineural hearing loss [9]. Prospective dosimetric work demonstrated a dose–response relationship between inner-ear dose and hearing deterioration, with clinically relevant hearing loss appearing predominantly above 40–45 Gy, but measurable threshold shifts occurring at lower exposures [10]. Comprehensive reviews of radiotherapy-induced ear toxicity emphasize that conductive, sensorineural, and mixed patterns may all occur and that cochlear dose is the dominant modifiable determinant [11].

When irradiation and platinum are combined, auditory toxicity is amplified. A systematic review of sensorineural hearing loss after CRT and radiotherapy alone for HNC reported wide variation in incidence, between approximately 17% and 88%, reflecting heterogeneous definitions, audiometric protocols, and follow-up schedules [12]. Analyses attempting to separate the relative contributions of the two modalities indicate that cisplatin-based chemotherapy and cochlear radiation dose contribute jointly and approximately additively to hearing deterioration [13], and randomized data confirm substantial audiometric toxicity, even with modern delivery techniques [14]. Nevertheless, most available cohorts predate contemporary intensity-modulated planning, and real-world data describing early auditory outcomes under current cochlear-sparing practice remain scarce.

The present study addresses this gap using a contemporary, consecutive, single-center cohort treated with modern (chemo)radiotherapy in 2024–2025. The novelty of our approach lies in combining routinely recorded, plan-derived cochlear maximum point doses with early clinical hearing outcomes, and in applying complementary statistical techniques—penalized multivariable regression, dose–response modeling with restricted cubic splines, and exploratory discrimination and decision-curve analyses—to a pragmatic clinical dataset. Our objectives were (i) to quantify the frequency of early post-treatment hearing changes; (ii) to compare patients treated with concurrent CRT versus radiotherapy alone; and (iii) to characterize the relationship between cochlear radiation exposure and early hearing change, including its shape, its robustness to adjustment for tumor site and other covariates, and its behavior in sensitivity analyses.

## 2. Materials and Methods

### 2.1. Study Design and Population

We performed a retrospective, observational cohort study at the tertiary academic otolaryngology service affiliated with the “Victor Babes” University of Medicine and Pharmacy, Timisoara, Romania. Consecutive adult patients with histologically confirmed head and neck malignancies who initiated external-beam radiotherapy between November 2024 and September 2025 and who were referred for otolaryngologic and audiologic evaluation were identified from the departmental treatment registry. The study followed the principles of the Declaration of Helsinki, and ethical approval was received from the local Institutional Ethics Committee. Informed consent was obtained from all participants involved in the study.

Demographic, oncologic, treatment, and audiologic data were abstracted from electronic medical records, radiotherapy treatment plans, and audiology charts using a standardized extraction form. Tumor subsite, clinical TNM category, p16 status where available, prior surgery, and systemic treatment details were retrieved from the multidisciplinary tumor board documentation. All records were reviewed by two investigators, discrepancies were resolved by consensus, and the final analysis dataset comprised 70 patients.

### 2.2. Eligibility Criteria and Group Allocation

Patients were eligible if they were aged 18 years or older, had a primary malignant tumor of the head and neck region (including salivary gland and sinonasal primaries and cervical metastases of unknown primary), received at least one fraction of external-beam radiotherapy with a recorded treatment plan including bilateral cochlear-dose metrics, and had a documented clinical assessment of hearing status before and after treatment. Patients without retrievable planning data or without any post-treatment hearing documentation were excluded, as were patients treated exclusively with brachytherapy or systemic therapy.

Group allocation reflected the treatment actually delivered: the chemoradiotherapy (CRT) group comprised patients who received any concurrent systemic therapy during irradiation (weekly cisplatin 40 mg/m^2^ as the departmental standard, or carboplatin—area under the curve (AUC) 1.5–2 weekly or AUC 5 every three weeks—in cases of cisplatin ineligibility because of renal impairment, hearing impairment, neuropathy, or poor performance status; two patients who started cisplatin and were switched to carboplatin after the first cycle because of renal toxicity were retained in the CRT group but not in agent-specific subgroups), whereas the radiotherapy-alone (RT) group received irradiation without concurrent systemic agents. Induction chemotherapy (predominantly docetaxel–cisplatin–5-fluorouracil or cisplatin–5-fluorouracil, with cisplatin 75–100 mg/m^2^ per cycle for a median of three cycles) was recorded separately and did not determine group assignment. For every patient, the cumulative cisplatin dose (mg/m^2^) was calculated as the sum of induction and concurrent cisplatin actually administered, and carboplatin exposure was recorded as the number of cycles. Radiotherapy was delivered with volumetric techniques using simultaneous integrated boost or sequential schedules; for simultaneous integrated boost prescriptions, the highest dose level was analyzed, and sequential boosts were summed.

### 2.3. Variables, Outcomes, and Data Collection

The primary outcome was any clinically documented post-treatment hearing change, defined as a new or worsened subjective hearing complaint corroborated by the treating otolaryngologist and/or audiometric deterioration on available follow-up testing, recorded as a binary variable. Audiometry, when performed, consisted of pure-tone air- and bone-conduction thresholds at 0.25–8 kHz; otoscopy was documented at every otolaryngologic visit and tympanometry when clinically indicated. Audiometric deterioration was assessed only in patients with paired (pre- and post-treatment) audiograms and was defined according to the American Speech-Language-Hearing Association (ASHA) criteria for ototoxicity monitoring: a ≥20 dB threshold increase at any single frequency, a ≥10 dB increase at two adjacent frequencies, or loss of response at three consecutive frequencies at which responses were previously obtained, in either ear [15]; the shift in the pure-tone average (0.5–4 kHz) of the worse ear was recorded in addition. For audiometrically documented events, the pattern of hearing change was classified as sensorineural, conductive, or mixed on the basis of the air–bone gap (≥15 dB averaged over 0.5–2 kHz indicating a conductive component), tympanometry (type B or C), and otoscopic findings. For events documented by symptoms alone, otoscopic and tympanometric findings were used only to flag a possible conductive component (middle-ear effusion, tympanic membrane retraction, or occlusive external-canal changes); these events could not be attributed specifically to cochlear injury and were analyzed as a composite “clinically apparent hearing change”. Pre-treatment otolaryngologic evaluation was part of the routine departmental pathway for every patient starting radiotherapy, and a post-treatment otolaryngologic review was scheduled routinely 4–8 weeks after the last fraction; audiometry, however, was not protocolized and was performed according to clinician judgment, patient complaint, and audiology capacity, so that repeat audiometry was predominantly symptom-driven. “Early” hearing change refers to any change documented between radiotherapy completion and the data lock (15 December 2025), with the interval from the last fraction to the first post-treatment hearing assessment and the total follow-up duration (from the last fraction to the last contact or death) recorded for each patient. Secondary outcomes included performance of audiometry at treatment initiation and repeat audiometry during follow-up, treatment interruption, treatment abandonment, and death during the observation period. Pre-treatment hearing loss was defined as any documented subjective or audiometric hearing impairment before the first radiotherapy fraction.

Exposure variables comprised age; residence (rural/urban); tumor subsite grouped as oropharynx, larynx/hypopharynx, and other sites; clinical T4 category; nodal positivity; prescribed radiotherapy dose and number of fractions; overall treatment time (OTT, days from first to last fraction); induction chemotherapy; concurrent agent and number of cycles; cumulative cisplatin dose (induction plus concurrent); and prior oncologic surgery. Cochlear exposure was quantified as the maximum point dose (Dmax) to each cochlea extracted from the approved treatment plan, from which we derived the bilateral mean cochlear Dmax (average of the two sides), the higher-ear Dmax (maximum of the two sides), and a binary indicator of cochlear Dmax ≥45 Gy. Because the QUANTEC recommendation refers to the mean cochlear dose [9], the mean dose to each cochlea (Dmean) was additionally retrieved from the archived dose–volume histograms of the approved plans, and compliance with the QUANTEC thresholds (≤45 Gy and ≤35 Gy where cochlear sparing is feasible) was evaluated per cochlea using Dmean. Dmax was retained as the prespecified primary exposure metric, with Dmean analyses reported as a sensitivity analysis. Curative intent was defined as a prescribed dose ≥50 Gy in the absence of documented palliative intent.

### 2.4. Statistical Analysis

Continuous variables are summarized as medians with interquartile ranges (IQR) because Shapiro–Wilk testing and inspection of distributions indicated non-normality for dosimetric and time variables; categorical variables are presented as counts and percentages. Between-group comparisons used the Mann–Whitney U test for continuous variables, with the Hodges–Lehmann estimator reported as the effect size for location shift, and the Fisher exact test (or the chi-square test for variables with more than two categories) for categorical variables. Effect sizes for binary outcomes are expressed as odds ratios (OR) with 95% confidence intervals (CI) computed by the Woolf method with Haldane–Anscombe correction, and as absolute risk differences with Wald 95% CI. Monotonic associations were quantified with Spearman rank correlation coefficients.

Given the modest number of events, multivariable logistic regression used Firth penalized likelihood, which reduces small-sample bias and accommodates sparse data; the adjusted model included age (per decade), pre-treatment hearing loss, concurrent CRT, and mean cochlear Dmax (per 10 Gy) as prespecified clinically motivated covariates. The shape of the dose–response relationship was examined with a restricted cubic spline with three degrees of freedom in a binomial generalized linear model, with likelihood-ratio tests for overall association and non-linearity. In response to peer review, a post hoc parsimonious Firth model was fitted that included tumor site (oropharynx vs. other sites), mean cochlear Dmax (per 10 Gy), and concurrent CRT, in order to assess whether the cochlear-dose association persisted after accounting for site; a likelihood-ratio test compared this model with a site-only model. Site-specific cochlear doses were compared with the Kruskal–Wallis test, and within-site Spearman correlations between cochlear dose and hearing change were computed. A Cochran–Armitage test evaluated the trend in outcome rates across cochlear-dose tertiles. Discrimination (area under the receiver operating characteristic curve (AUC) with 2000-sample bootstrap percentile 95% CI, cutoffs selected by the Youden index) and decision-curve analysis were prespecified but are regarded as exploratory because the study was not designed to derive a dose threshold.

Missing data were not imputed; analyses used all available cases, and the denominator is reported for every estimate (cochlear dosimetry was unavailable for one patient and the number of concurrent cycles for two patients). Robustness was addressed through prespecified sensitivity analyses restricting the cohort to curative-intent treatment, excluding deaths and treatment abandonment, excluding interrupted courses, excluding carboplatin-only patients, and restricting to patients without baseline hearing loss, complemented by an E-value calculation for unmeasured confounding; the univariable and adjusted dose models were also repeated with mean cochlear Dmean in place of Dmax. All tests were two-sided with statistical significance set at *p* < 0.05; exact *p*-values are reported without adjustment for multiplicity given the exploratory design, and all computations were performed in Python 3.12 (pandas 2.x, https://pandas.pydata.org; SciPy 1.x, https://scipy.org; statsmodels 0.14, https://www.statsmodels.org; scikit-learn 1.x, https://scikit-learn.org).

## 3. Results

### 3.1. Patient, Tumor, and Treatment Characteristics

The cohort included 70 patients: 48 (68.6%) received concurrent chemoradiotherapy (CRT) and 22 (31.4%) radiotherapy (RT) alone (Table 1). RT-alone patients were older (median 72.0 vs. 68.0 years, *p* = 0.014), whereas node-positive disease (66.7% vs. 27.3%, *p* = 0.004) and induction chemotherapy (35.4% vs. 9.1%, *p* = 0.023) were more frequent in the CRT group. Other baseline characteristics, including T4 category, prior surgery, and pre-treatment hearing loss, did not differ significantly.

Radiotherapy and dosimetric characteristics are shown in Table 2. Curative-intent treatment was more frequent with CRT (97.9% vs. 81.8%, *p* = 0.031), and RT-alone patients received fewer fractions and shorter treatment courses. Median bilateral mean cochlear Dmax was higher with CRT than RT alone (6.3 vs. 2.0 Gy, *p* = 0.001), as was bilateral mean cochlear Dmean (4.1 vs. 1.3 Gy, *p* = 0.001). Dmean and Dmax were strongly correlated (Spearman ρ = 0.97, *p* < 0.001). A cochlear Dmax ≥45 Gy occurred in two of 69 evaluable patients (2.9%), but no patient exceeded the QUANTEC mean-dose constraint of 45 Gy in either cochlea, and the per-cochlea Dmean exceeded 35 Gy in only one patient (38.6 Gy; the two patients with Dmax ≥45 Gy had higher-ear Dmean values of 38.6 and 29.4 Gy). Systemic therapy exposure was heterogeneous. Among the 48 CRT patients, 41 received concurrent cisplatin (median five cycles, IQR 4–6; median cumulative concurrent dose 200 mg/m^2^, IQR 160–240), five received carboplatin only (median three cycles, IQR 2–5), and two were switched from cisplatin to carboplatin after the first cycle. Cisplatin-based induction chemotherapy was given to 19 patients (17 CRT, two RT alone; median induction cisplatin dose 225 mg/m^2^, IQR 150–300), of whom 14 subsequently received concurrent cisplatin, three concurrent carboplatin, and two radiotherapy alone. Overall, 48 patients had any cisplatin exposure (median cumulative dose 240 mg/m^2^, IQR 200–400; 17 patients ≥ 300 mg/m^2^) and 22 had none.

**Figure 1:**
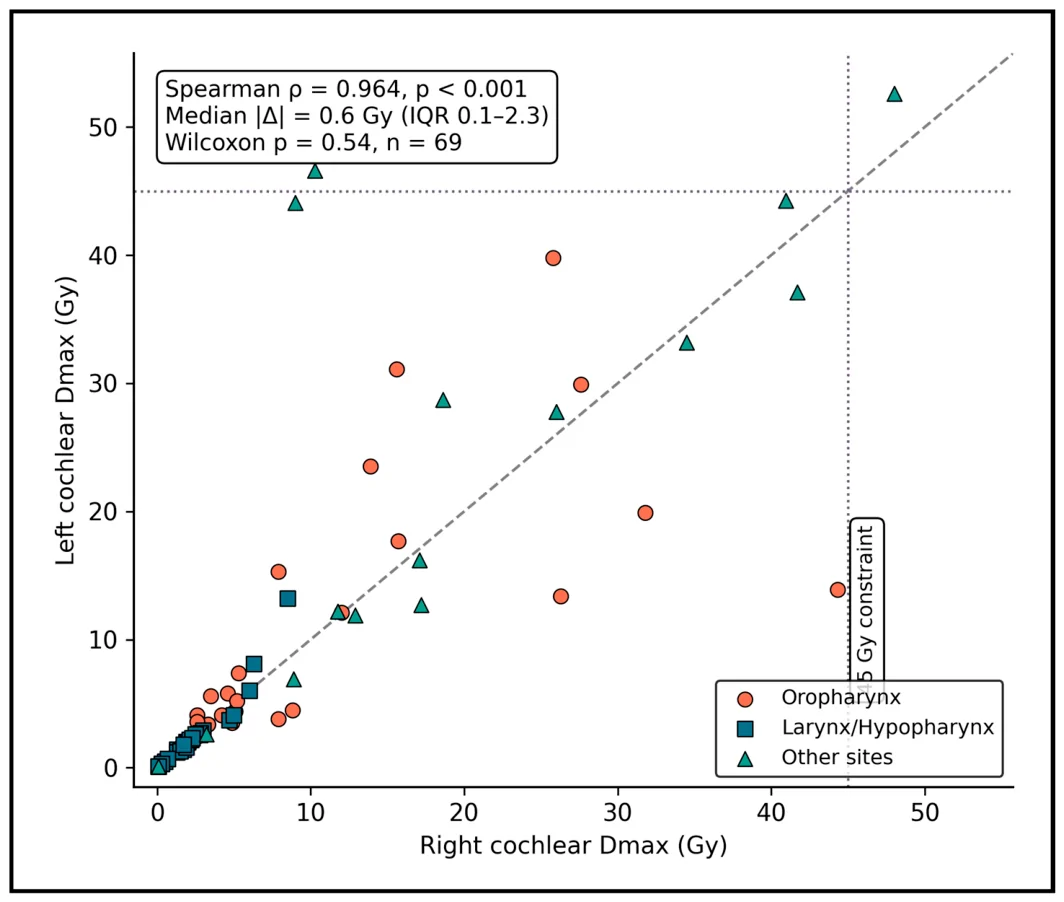
Interaural cochlear-dose symmetry.

Interaural dose symmetry is shown in Figure 1. Left and right cochlear Dmax were strongly correlated (Spearman ρ = 0.964, *p* < 0.001; *n* = 69), with no systematic lateralization (Wilcoxon *p* = 0.54). The median absolute interaural difference was 0.6 Gy (IQR 0.1–2.3), although larger differences occurred at higher exposures.

### 3.2. Hearing Outcomes

Primary and secondary outcomes are summarized in Table 3. Hearing change occurred in 38/70 patients (54.3%): 28/48 (58.3%) after CRT and 10/22 (45.5%) after RT alone (OR 1.68, 95% CI 0.61–4.49; *p* = 0.439). Among patients without pre-treatment hearing loss, new hearing change occurred in 6/15 CRT and 1/3 RT patients. All patients underwent the routine pre-treatment and post-treatment otolaryngologic reviews. Audiometry at treatment initiation was performed in 31/70 patients (44.3%) and repeat audiometry in 10/70 (14.3%); all 10 patients with repeat audiometry also had a baseline audiogram, so paired audiograms were available in 10/70 (14.3%). Repeat audiometry had been prompted by a new hearing complaint in eight of these 10 patients. Audiometric deterioration according to the ASHA criteria was present in 7/10 patients with paired audiograms (70.0%; 6/8 CRT and 1/2 RT alone), and a ≥10 dB shift in the worse-ear pure-tone average in 5/10; all seven patients with audiometric deterioration had also reported a new complaint, whereas one patient with a corroborated complaint but no audiometric deterioration was classified as hearing change on clinical grounds. Consequently, seven of the 38 hearing-change events (18.4%) were audiometrically confirmed, and 31 (81.6%) rested on a clinician-corroborated subjective complaint alone (Table 4). Of the seven audiometrically documented events, four were sensorineural (high-frequency bone-conduction threshold shifts without an air–bone gap), two mixed, and one conductive (air–bone gap ≥15 dB with a type B tympanogram and middle-ear effusion on otoscopy). Among the 31 symptom-only events, otoscopy showed findings compatible with a conductive component in 11 (35.5%), and tympanometry, available in nine of these patients, was type B or C in five. Overall, a conductive or mixed component was therefore documented or suspected in 14/38 events (36.8%), a purely sensorineural change was confirmed in only 4/38 (10.5%), and the pattern was indeterminate in the remaining 20 (52.6%). The first post-treatment hearing assessment took place a median of 5.1 weeks after the last fraction (IQR 3.4–8.6; range 1.0–19.3), and the median follow-up from radiotherapy completion was 3.4 months (IQR 1.9–5.6; range 0.7–10.8); 24 patients (34.3%) were followed for less than 2 months, 29 (41.4%) for 2–6 months, and 17 (24.3%) for more than 6 months.

Continuous characteristics by hearing outcome are presented in Table 5. Median cochlear Dmax was higher in patients with hearing change (5.5 vs. 2.5 Gy, *p* = 0.045), whereas age, prescribed RT dose, overall treatment time, and concurrent-cycle number were not significantly different. Similar differences were observed for the higher-ear cochlear Dmax, whereas the difference in mean cochlear Dmean fell short of statistical significance (3.6 vs. 1.7 Gy, *p* = 0.052).

**Figure 2:**
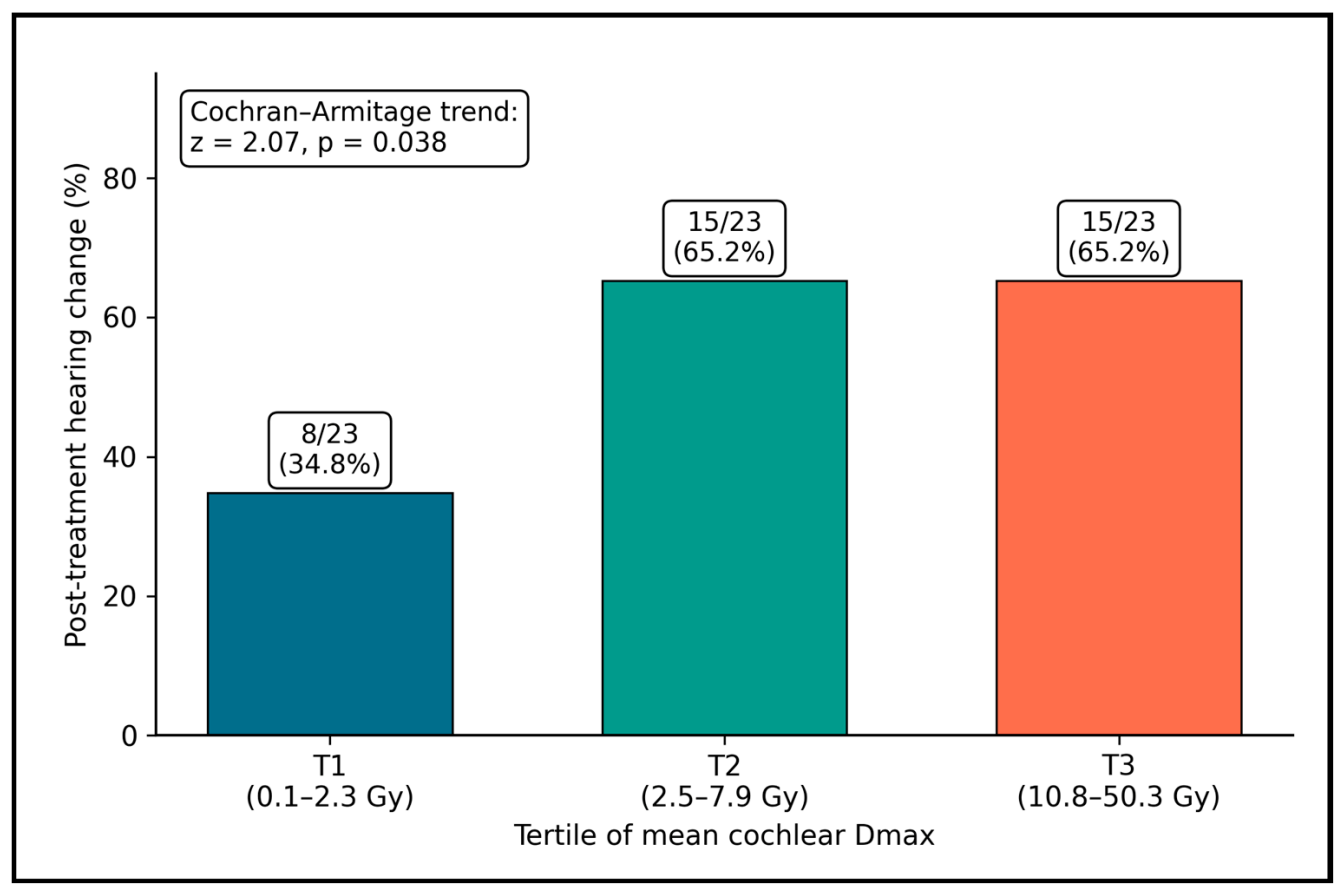
Hearing change by cochlear-dose tertile.

Figure 2 shows hearing-change rates across tertiles of mean cochlear Dmax. Rates were 34.8% (8/23), 65.2% (15/23), and 65.2% (15/23) in the first, second, and third tertiles, respectively, with a significant ordered trend (Cochran–Armitage z = 2.07, *p* = 0.038).

### 3.3. Subgroup and Correlation Analyses

Subgroup results are shown in Table 6. Tumor site was associated with hearing change (*p* = 0.017), with the highest rate in oropharyngeal tumors (74.1%). Differences by age, residence, T4 category, nodal status, baseline hearing loss, prior surgery, induction chemotherapy, and treatment group were not statistically significant. Cumulative cisplatin exposure was likewise not associated with hearing change: 11/17 (64.7%) patients with ≥300 mg/m^2^, 17/31 (54.8%) with 1–299 mg/m^2^, and 10/22 (45.5%) with no cisplatin exposure developed hearing change (chi-square *p* = 0.49; Spearman ρ = 0.16, *p* = 0.19).

Because tumor site was associated with hearing change, cochlear exposure was examined by site (Table 7). Oropharyngeal primaries received markedly higher cochlear doses (median cochlear Dmax 9.8 Gy) than laryngeal/hypopharyngeal primaries (1.9 Gy), with other sites intermediate (6.5 Gy; Kruskal–Wallis *p* < 0.001), so that site and cochlear dose were strongly collinear. Within each site, cochlear dose was not correlated with hearing change (Spearman ρ 0.11–0.19, all *p* > 0.4), and the hearing-change rate in oropharyngeal tumors remained high irrespective of whether cochlear Dmax was below or above the site-specific median (9/13 vs. 11/14).

Table 8 summarizes Spearman correlations. Mean cochlear Dmax was positively correlated with hearing change (ρ = 0.244, *p* = 0.043). Age, RT dose, overall treatment time, and concurrent-cycle number were not significantly correlated with the outcome.

**Figure 3:**
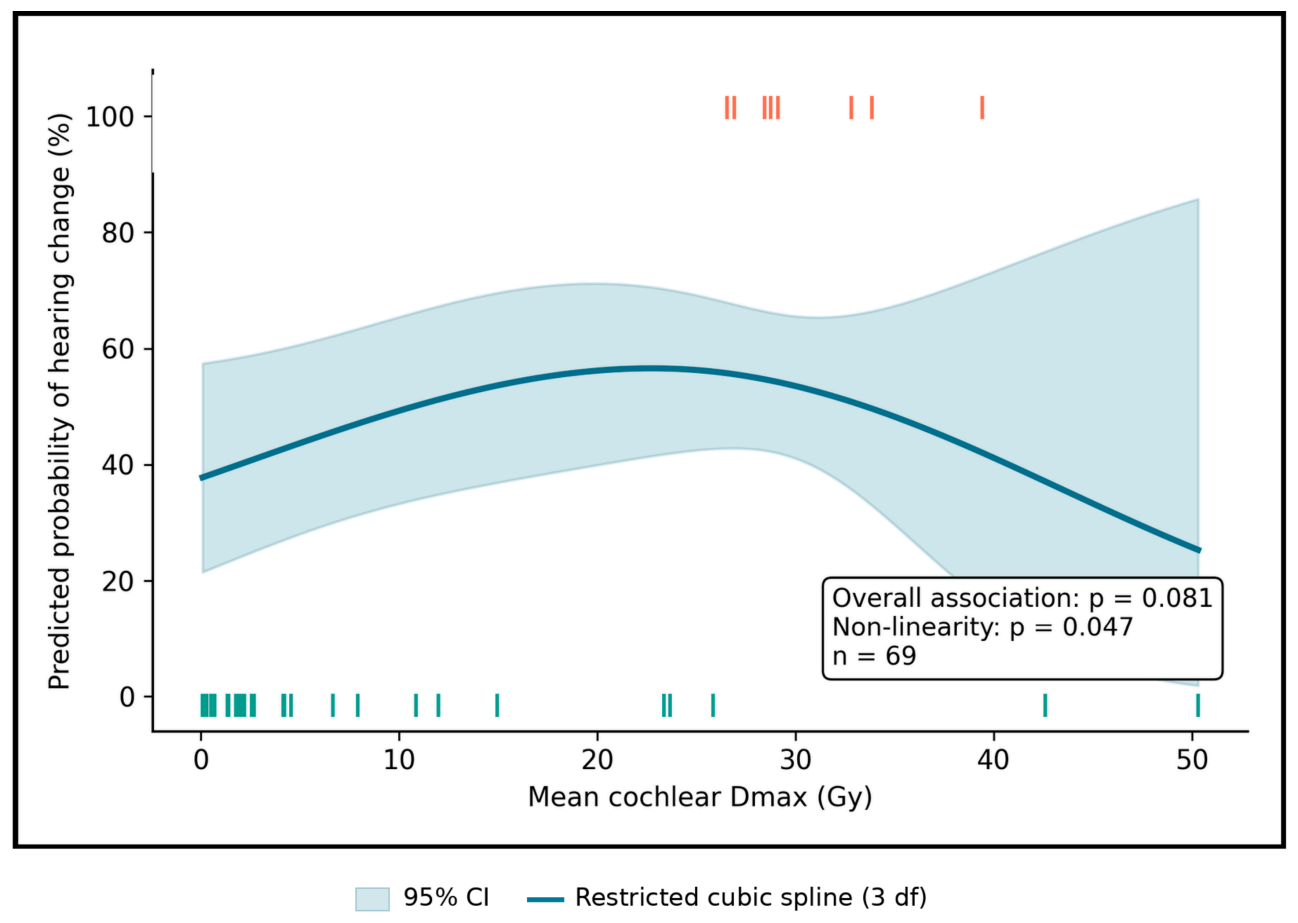
Cochlear dose–response curve. The solid line shows the restricted cubic spline (3 degrees of freedom) estimate of the probability of hearing change as a function of the mean cochlear maximum point dose (Dmax), and the shaded band its 95% confidence interval (CI); the tick marks at the top and bottom of the plot indicate individual patients with and without hearing change, respectively.

The dose–response pattern is shown in Figure 3. The estimated probability of hearing change increased through approximately 20–25 Gy and then flattened, with wider confidence intervals at higher doses. The overall spline association was *p* = 0.081 and the non-linearity test was *p* = 0.047 (*n* = 69).

### 3.4. Multivariable Modeling

Firth logistic regression estimates are presented in Table 9. In the adjusted model (*n* = 69), the ORs were 1.28 per decade of age (95% CI 0.70–2.33; *p* = 0.425), 2.04 for pre-treatment hearing loss (0.52–8.11; *p* = 0.309), 1.66 for concurrent CRT (0.54–5.10; *p* = 0.373), and 1.31 per 10 Gy increase in mean cochlear Dmax (0.82–2.08; *p* = 0.253). Thus, the univariable association between cochlear dose and hearing change was not statistically significant after adjustment. Substituting mean cochlear Dmean for Dmax gave similar estimates (unadjusted OR 1.29 per 10 Gy, 95% CI 0.76–2.19, *p* = 0.345; adjusted OR 1.38 per 10 Gy, 95% CI 0.80–2.39, *p* = 0.246).

In the post hoc parsimonious model that accounted for tumor site (Table 10), oropharyngeal primary was the only variable associated with hearing change (OR 4.02, 95% CI 1.28–12.6; *p* = 0.017), whereas the OR for mean cochlear Dmax was attenuated to 1.09 per 10 Gy (95% CI 0.66–1.80; *p* = 0.735) and that for concurrent CRT was attenuated to 1.21 (95% CI 0.38–3.85; *p* = 0.748). Adding cochlear dose to a site-only model did not improve fit (likelihood-ratio *p* = 0.64). A two-variable model containing site and dose only gave the same picture (oropharynx OR 3.86, 95% CI 1.27–11.7, *p* = 0.017; Dmax OR 1.12 per 10 Gy, 95% CI 0.69–1.82, *p* = 0.645).

Exploratory discrimination and decision-curve analyses were performed. Mean cochlear Dmax alone discriminated hearing change only modestly (AUC 0.642, 95% CI 0.505–0.772; Dmean 0.636, 95% CI 0.498–0.767); the adjusted model reached an apparent AUC of 0.669 (95% CI 0.534–0.788), and the decision curve favored an assess-all strategy across clinically relevant low threshold probabilities. The Youden-derived cutoff for mean cochlear Dmax (≥2.75 Gy; sensitivity 76.3%, specificity 54.8%) is reported for completeness only and is not proposed as a dose constraint.

### 3.5. Sensitivity Analyses

Sensitivity analyses are summarized in Table 11. The OR for CRT versus RT remained above 1.0 across all restricted analyses, ranging from 1.52 to 2.32, but no comparison reached statistical significance. In patients without baseline hearing loss, the OR was 1.33 (95% CI 0.12–10.89). The E-value for the primary point estimate was 1.92.

## 4. Discussion

The present study demonstrates that early, clinically documented hearing change is common after contemporary head and neck (chemo)radiotherapy, occurring in 54.3% of patients. This frequency lies within the broad range reported in previous radiation and combined-modality cohorts [16,17]. Although the crude rate was higher after CRT than RT alone, the difference was imprecisely estimated and not statistically significant. By contrast, the clearest univariable signals were cochlear radiation exposure and tumor subsite, consistent with prior dosimetric series in which proximity of pharyngeal targets to the auditory apparatus was associated with greater hearing deterioration [18,19]. However, the cochlear-dose association was not statistically significant in the adjusted Firth model (OR 1.31 per 10 Gy, 95% CI 0.82–2.08) and was attenuated almost to the null once tumor site was taken into account (OR 1.09 per 10 Gy), whereas oropharyngeal primary remained associated with hearing change. The two exposures are strongly collinear in this cohort—oropharyngeal targets delivered a fivefold higher median cochlear dose than laryngeal targets—so our data cannot separate a dose effect from a site effect, and the univariable dose gradient should not be read as evidence of an independent effect of low-dose cochlear exposure. Population studies of cisplatin-treated survivors likewise show that clinically important hearing impairment is common when it is measured systematically [20,21].

The low-dose gradient observed in the univariable analyses deserves cautious interpretation. Hearing-change rates increased across cochlear-dose tertiles, mean cochlear Dmax correlated with the outcome, and the spline analysis suggested that much of the early increase occurred within the lower portion of the observed dose range. The exploratory ROC-derived cutoff near 3 Gy must not be interpreted as a biological safety threshold or translated into a clinical dose constraint: its discriminatory performance was modest (AUC 0.642) with wide confidence intervals, the study was not designed to derive a dose threshold, and the association from which it was derived did not persist after adjustment. At most, the univariable pattern indicates that clinically apparent early changes were not confined to patients approaching the conventional 35–45 Gy planning limits described in the literature [16,17]. Importantly, no patient in this cohort exceeded the QUANTEC mean cochlear-dose constraint of 45 Gy, and only one exceeded 35 Gy, so the cohort offers no information on the benefit of dose reduction below current constraints. Taken together with the predominance of symptom-based rather than audiometrically confirmed events, these data do not show that further cochlear-dose reduction would improve hearing outcomes; they indicate instead that early hearing change occurs frequently at low cochlear doses and that its causes—cochlear, middle-ear, and systemic—remain to be disentangled in prospective studies with protocolized audiometry.

The treatment-modality results are also compatible with a multifactorial model of ototoxicity. Weekly cisplatin, the predominant regimen in this cohort, has been associated with less ototoxicity than higher-dose three-weekly schedules [22,23], which may help explain the modest CRT–RT difference despite the known cochlear toxicity of platinum. At the same time, platinum injury can accumulate and persist well beyond treatment [24], so our short follow-up is likely to underestimate later divergence between treatment groups. Differences in ototoxicity definitions further complicate direct comparison across studies [25,26,27]. Systemic therapy exposure in this cohort was also heterogeneous: 41 patients received concurrent cisplatin, five carboplatin only, two were switched from cisplatin to carboplatin, and 19 received cisplatin-based induction, so that cumulative cisplatin exposure ranged from none to more than 400 mg/m^2^ and was unevenly distributed between treatment groups and across tumor sites (induction was given predominantly for pharyngeal primaries). Carboplatin at the schedules used in HNC is considerably less ototoxic than cisplatin [7,25], while induction adds cumulative platinum before irradiation and may itself induce threshold shifts that precede the baseline assessment; both are therefore potential confounders of the CRT–RT comparison and of the dose association. Cumulative cisplatin dose was not significantly associated with hearing change, and the exclusion of carboplatin-only patients did not alter the CRT–RT estimate, but the small numbers in each exposure category preclude any firm separation of the contribution of individual agents. Moreover, the systemic treatment landscape of HNC is becoming more complex, with immune checkpoint inhibition and biologically stratified strategies increasingly integrated alongside, or in place of, platinum-based regimens [28]; as regimens diversify, attributing hearing toxicity to specific agents will require prospective, agent-specific ototoxicity monitoring rather than retrospective group comparisons. Because our primary endpoint combined clinician-documented symptoms with audiometry when available, it preferentially captures functionally apparent changes rather than subtle high-frequency threshold shifts; moreover, only seven of 38 events were audiometrically confirmed, and a conductive or mixed component was documented or suspected in more than one-third of events, so the endpoint cannot be equated with cochlear injury.

The dosimetric findings have practical implications for treatment planning. Left and right cochlear doses were highly correlated for most patients, supporting use of a bilateral patient-level summary in population analyses, but occasional marked interaural differences remained at higher exposures. Such asymmetry is clinically relevant in lateralized or skull-base targets and argues for continued per-ear review during planning. The high hearing-change rate in oropharyngeal disease is, at least in part, explained by treatment geometry: oropharyngeal primaries received a median cochlear Dmax of 9.8 Gy versus 1.9 Gy for laryngeal/hypopharyngeal primaries, and within-site dose was no longer correlated with hearing changes. Oropharyngeal and nasopharyngeal irradiation also exposes the Eustachian tube and middle ear, so part of the site effect may reflect radiation-related middle-ear dysfunction rather than cochlear injury, in keeping with the otoscopic and tympanometric findings in our symptom-only events. These observations still support careful cochlear contouring and per-ear review in pharyngeal tumors, where target geometry can bring one or both cochleae closer to the high-dose volume [18,19], but they do not establish that cochlear dose itself drives early hearing change at the low doses observed here.

Finally, the study highlights an important surveillance gap. Fewer than half of patients had audiometry at treatment initiation, and only a small minority underwent repeat testing, a pattern consistent with implementation barriers reported in ototoxicity-monitoring programs [29,30]. Without serial audiograms, early detection, grading, counseling, and rehabilitation are constrained, despite the recognized effects of hearing loss on communication and quality of life [31,32]. The multivariable model showed only moderate discrimination, and the decision curve favored an assess-all strategy across low threshold probabilities; therefore, risk prediction should not be used to exclude patients from baseline monitoring. Where audiology capacity is limited, risk models might eventually help prioritize intensified follow-up, but the models explored here are unvalidated and of unproven clinical usefulness, and protocolized baseline and post-treatment testing remain the appropriate default. The consistent direction of the sensitivity analyses supports further prospective evaluation in larger cohorts.

### Study Limitations

Several limitations must be acknowledged. This was a retrospective, single-center study of 70 patients, and the modest number of events limited statistical power, produced wide confidence intervals around all effect estimates, and precluded adjustment for more than four covariates; the possibility of residual and unmeasured confounding—by comorbidity, ototoxic co-medication, noise exposure, or smoking—remains, as quantified by the E-value analysis. The primary outcome combined clinician-documented symptomatic change with audiometry where available; because baseline audiometry was performed in fewer than half of patients and paired audiograms were available in only 10, subtle high-frequency threshold shifts were certainly under-ascertained, and the outcome should be interpreted as clinically apparent early hearing change rather than criterion-based sensorineural loss. Because repeat audiometry was predominantly symptom-driven, the proportion of events with audiometric confirmation is not representative of the cohort, and asymptomatic threshold shifts in patients who were not re-tested were missed. Conductive and sensorineural changes could be distinguished only in the seven audiometrically documented events; for the remaining events, otoscopy and limited tympanometry allowed a conductive component to be suspected but not excluded, so the hearing changes reported here cannot be attributed specifically to cochlear injury. Tumor site and cochlear dose were strongly collinear, and the modest sample size did not permit a fully adjusted model containing all candidate covariates; the parsimonious site-adjusted model was fitted post hoc and should be regarded as exploratory. Cochlear exposure was characterized primarily by the planned maximum point dose; mean cochlear doses retrieved from the dose–volume histograms gave concordant results, but neither metric accounts for equivalent dose in 2 Gy fractions, and delivered-dose deviations were not captured. Follow-up was short (median 3.4 months from the last fraction, with one-third of patients followed for less than 2 months), so the findings pertain only to early changes, and late progressive ototoxicity is not represented. The predominance of elderly patients with baseline hearing impairment, the single-institution referral pattern, and local treatment policies may limit generalizability, and the apparent model performance was not externally validated. Prospective studies with protocolized serial audiometry, extended follow-up, and multicenter recruitment are needed to validate these findings.

## 5. Conclusions

Early, clinically apparent hearing changes were common after modern (chemo)radiotherapy for head and neck cancer in this consecutive single-center cohort, affecting more than half of patients even though no patient exceeded the QUANTEC mean cochlear-dose constraint. Cochlear dose was associated with hearing change in univariable analyses only; the association was not statistically significant after adjustment and was largely accounted for by tumor site, with oropharyngeal primaries receiving the highest cochlear doses and showing the highest rate of hearing change. Because paired audiometry was available in a small minority and a conductive component could not be excluded in many events, the observed changes cannot be attributed specifically to cochlear injury, and these data do not show that further cochlear-dose reduction below current constraints would improve hearing outcomes. The principal practical implication is that baseline and follow-up audiometry, with air- and bone-conduction thresholds and tympanometry, deserve systematic integration into head and neck cancer care pathways; the exploratory dose cutoff and risk models reported here should not be used clinically until validated prospectively. Prospective studies with protocolized serial audiometry, longer follow-up, and adequate power to separate the effects of tumor site, cochlear dose, and systemic therapy are warranted.

## Figures and Tables

**Table 1:** Baseline demographic and disease characteristics by treatment group.

Characteristic	CRT (*n* = 48)	RT Alone (*n* = 22)	*p*-Value
Age, years, median (IQR)	68.0 (59.5–71.0)	72.0 (65.5–78.8)	0.014
Age ≥65 years, *n* (%)	33 (68.8)	16 (72.7)	0.787
Rural residence, *n* (%)	22 (45.8)	10 (45.5)	1.000
Tumor site, *n* (%)			0.050
Oropharynx	18 (37.5)	9 (40.9)	
Larynx/hypopharynx	16 (33.3)	12 (54.5)	
Other sites	14 (29.2)	1 (4.5)	
Clinical T4 category, *n* (%)	13 (27.1)	4 (18.2)	0.553
Node-positive disease, *n* (%)	32 (66.7)	6 (27.3)	0.004
Pre-treatment hearing loss, *n* (%)	33 (68.8)	19 (86.4)	0.148
Prior oncologic surgery, *n* (%)	7 (14.6)	3 (13.6)	1.000
Induction chemotherapy, *n* (%)	17 (35.4)	2 (9.1)	0.023

CRT, concurrent chemoradiotherapy; RT, radiotherapy; IQR, interquartile range. Other sites include oral cavity, nasopharynx, salivary gland, paranasal sinus, and unknown primary. *p*-values from Mann–Whitney U test (continuous), Fisher exact test (binary), or chi-square test (multicategory) are shown.

**Table 2:** Radiotherapy prescription and cochlear dosimetry by treatment group.

Parameter	CRT (*n* = 48)	RT Alone (*n* = 22)	*p*-Value
Prescribed dose, Gy, median (IQR)	70.0 (70.0–70.0)	70.0 (60.8–70.0)	0.003
Fractions, median (IQR)	35 (35–35)	35 (28.5–35)	0.002
Curative-intent treatment, *n* (%)	47 (97.9)	18 (81.8)	0.031
Overall treatment time, days, median (IQR)	50.0 (47.0–52.0)	46.5 (38.5–49.8)	0.003
Left cochlear Dmax, Gy, median (IQR)	5.9 (2.6–16.6)	2.0 (0.5–3.8)	0.001
Right cochlear Dmax, Gy, median (IQR)	6.2 (2.6–16.1)	2.0 (0.5–5.1)	0.002
Mean cochlear Dmax, Gy, median (IQR)	6.3 (2.7–19.0)	2.0 (0.5–4.8)	0.001
Higher-ear cochlear Dmax, Gy, median (IQR)	7.0 (2.8–24.2)	2.0 (0.5–5.1)	0.001
Cochlear Dmax ≥ 45 Gy, *n* (%)	2 (4.2)	0 (0.0)	1.000
Left cochlear Dmean, Gy, median (IQR)	3.9 (1.6–11.2)	1.3 (0.4–2.6)	0.002
Right cochlear Dmean, Gy, median (IQR)	4.0 (1.7–11.8)	1.3 (0.4–3.4)	0.002
Mean cochlear Dmean, Gy, median (IQR)	4.1 (1.7–12.4)	1.3 (0.4–3.2)	0.001
Per-cochlea Dmean > 35 Gy, *n* (%)	1 (2.1)	0 (0.0)	1.000
Per-cochlea Dmean > 45 Gy, *n* (%)	0 (0.0)	0 (0.0)	—
Concurrent cisplatin cycles, median (IQR) ^1^	5.0 (4.0–6.0)	—	—
Cumulative concurrent cisplatin, mg/m^2^, median (IQR) ^1^	200 (160–240)	—	—
Concurrent carboplatin only, *n* (%)	5 (10.4)	—	—
Cisplatin switched to carboplatin, *n* (%)	2 (4.2)	—	—
Induction cisplatin dose, mg/m^2^, median (IQR) ^2^	225 (150–300)	150; 225	—
Cumulative cisplatin (induction + concurrent), mg/m^2^, median (IQR) ^3^	240 (200–400)	150; 225	—
Cumulative cisplatin ≥ 300 mg/m^2^, *n* (%)	17 (35.4)	0 (0.0)	0.001

CRT, concurrent chemoradiotherapy; RT, radiotherapy; IQR, interquartile range; Dmax, maximum point dose per treatment plan; Dmean, mean cochlear dose from the dose–volume histogram. ^1^ Among the 41 patients receiving concurrent cisplatin. ^2^ Among the 19 patients receiving induction chemotherapy (17 CRT; the two RT-alone values are shown individually). ^3^ Among the 48 patients with any cisplatin exposure (46 CRT; 2 RT alone, shown individually). Cochlear dosimetry is available for 69 patients.

**Table 3:** Primary and secondary outcomes by treatment group.

Outcome	CRT (*n* = 48)	RT Alone (*n* = 22)	Effect (95% CI)	*p*-Value
Post-treatment hearing change, *n* (%)	28 (58.3)	10 (45.5)	OR 1.68 (0.61–4.49)	0.439
Absolute risk difference, %	—	—	+12.9 (−12.2 to +37.9)	—
New-onset change if no baseline HL, *n*/*N* (%) ^1^	6/15 (40.0)	1/3 (33.3)	—	1.000
Audiometry at treatment initiation, *n* (%)	23 (47.9)	8 (36.4)	—	0.442
Repeat audiometry during follow-up, *n* (%)	8 (16.7)	2 (9.1)	—	0.488
Radiotherapy interruption, *n* (%)	4 (8.3)	2 (9.1)	—	1.000
Death during observation, *n* (%)	2 (4.2)	0 (0.0)	—	1.000
Treatment abandonment, *n* (%)	0 (0.0)	1 (4.5)	—	0.314

CRT, concurrent chemoradiotherapy; RT, radiotherapy; OR, odds ratio; CI, confidence interval; HL, hearing loss. ^1^ Among the 18 patients without pre-treatment hearing loss.

**Table 4:** Audiometric and otologic characterization of post-treatment hearing change and timing of assessment.

Characteristic	*n*/*N* (%) or Median (IQR)
Audiometry at treatment initiation	31/70 (44.3)
Repeat audiometry during follow-up	10/70 (14.3)
Paired (baseline and repeat) audiograms	10/70 (14.3)
Repeat audiometry prompted by a new complaint	8/10 (80.0)
Audiometric deterioration, ASHA criteria (paired audiograms)	7/10 (70.0)
Worse-ear pure-tone average shift ≥ 10 dB (paired audiograms)	5/10 (50.0)
Hearing-change events, total	38/70 (54.3)
Audiometrically confirmed	7/38 (18.4)
Clinician-corroborated complaint without paired audiometry	31/38 (81.6)
Pattern of audiometrically confirmed events (*n* = 7)	
Sensorineural	4/7 (57.1)
Mixed	2/7 (28.6)
Conductive	1/7 (14.3)
Tympanometry available	18/70 (25.7)
Type B or C tympanogram among hearing-change events with tympanometry	8/15 (53.3)
Otoscopic findings compatible with a conductive component (symptom-only events)	11/31 (35.5)
Conductive or mixed component documented or suspected (all events)	14/38 (36.8)
Purely sensorineural change confirmed (all events)	4/38 (10.5)
Pattern indeterminate (all events)	20/38 (52.6)
Interval from last fraction to first post-treatment hearing assessment, weeks	5.1 (3.4–8.6)
Follow-up from last fraction, months	3.4 (1.9–5.6)
<2 months	24/70 (34.3)
2–6 months	29/70 (41.4)
>6 months	17/70 (24.3)

ASHA, American Speech-Language-Hearing Association; IQR, interquartile range. ASHA criteria: ≥20 dB threshold increase at any single frequency, ≥10 dB at two adjacent frequencies, or loss of response at three consecutive previously responsive frequencies, in either ear. Conductive component was defined by an air–bone gap ≥15 dB (0.5–2 kHz), a type B or C tympanogram, and/or otoscopic evidence of middle-ear effusion, tympanic membrane retraction, or occlusive external-canal changes. Follow-up censored at the data lock (15 December 2025) or death.

**Table 5:** Patient and dosimetric variables according to post-treatment hearing status.

Variable, Median (IQR)	Hearing Change (*n* = 38)	No Change (*n* = 32)	HL Shift	*p*-Value
Age, years	69.0 (67.2–73.0)	68.0 (54.8–72.8)	2.0	0.419
Prescribed dose, Gy	70.0 (70.0–70.0)	70.0 (65.5–70.0)	0.0	0.394
Overall treatment time, days	49.0 (47.0–51.0)	49.0 (44.8–51.2)	0.0	0.933
Mean cochlear Dmax, Gy	5.5 (2.8–18.2)	2.5 (1.6–9.4)	1.95	0.045
Higher-ear cochlear Dmax, Gy	5.9 (2.8–22.1)	2.6 (1.6–10.5)	2.20	0.050
Left cochlear Dmax, Gy	5.5 (2.6–16.0)	2.5 (1.5–9.5)	1.90	0.051
Right cochlear Dmax, Gy	5.2 (2.6–13.7)	2.5 (1.6–8.9)	1.70	0.066
Mean cochlear Dmean, Gy	3.6 (1.8–11.9)	1.7 (1.0–6.2)	1.40	0.052
Concurrent chemotherapy cycles	4.5 (0.5–5.0)	3.0 (0.0–5.0)	0.0	0.152

IQR, interquartile range; Dmax, maximum point dose; HL Shift, Hodges–Lehmann estimate of the median difference between groups. *p*-values from Mann–Whitney U tests. Cochlear dosimetry was available for 69 patients; concurrent cycles for 68 patients.

**Table 6:** Post-treatment hearing change across prespecified subgroups.

Subgroup	*n*	Hearing Change, *n* (%)	*p*-Value ^1^
Age < 65 years	21	8 (38.1)	0.116
Age ≥ 65 years	49	30 (61.2)	
Rural residence	32	15 (46.9)	0.336
Urban residence	38	23 (60.5)	
Oropharynx	27	20 (74.1)	0.017
Larynx/hypopharynx	28	10 (35.7)	
Other sites	15	8 (53.3)	
Pre-treatment hearing loss	52	31 (59.6)	0.172
No pre-treatment hearing loss	18	7 (38.9)	
Concurrent cisplatin	41	24 (58.5)	0.205
Concurrent carboplatin only	5	4 (80.0)	
No concurrent chemotherapy	22	10 (45.5)	
Induction chemotherapy	19	10 (52.6)	1.000
No induction chemotherapy	51	28 (54.9)	

^1^ Fisher exact test (two categories) or chi-square test (three categories) comparing hearing-change rates within each stratification factor.

**Table 7:** Cochlear dose and post-treatment hearing change by tumor site.

Tumor Site	*n*	Mean Cochlear Dmax, Gy, Median (IQR)	Mean Cochlear Dmean, Gy, Median (IQR)	Hearing Change, *n* (%)	Within-Site Spearman ρ (*p*-Value) ^1^
Oropharynx	27	9.8 (4.1–24.6)	6.9 (2.8–16.5)	20 (74.1)	0.11 (0.58)
Larynx/hypopharynx	28	1.9 (0.6–3.1)	1.2 (0.4–2.0)	10 (35.7)	0.14 (0.49)
Other sites	15	6.5 (2.4–20.3)	4.4 (1.6–13.8)	8 (53.3)	0.19 (0.50)
*p*-Value ^2^		<0.001	<0.001	0.017	—

Dmax, maximum point dose; Dmean, mean cochlear dose; IQR, interquartile range. Cochlear dosimetry was available for 69 patients (one laryngeal/hypopharyngeal patient without dosimetry). ^1^ Spearman correlation between mean cochlear Dmax and hearing change within each site. ^2^ Kruskal–Wallis test for dose; chi-square test for hearing change.

**Table 8:** Spearman rank correlation matrix between quantitative variables and the hearing outcome.

Variable	Age	RT Dose	OTT	Mean Cochlear Dmax	Concurrent Cycles
RT dose	−0.047 (0.700)	—			
OTT	−0.140 (0.252)	0.672 (<0.001)	—		
Mean cochlear Dmax	−0.203 (0.095)	0.349 (0.003)	0.313 (0.009)	—	
Concurrent cycles	−0.179 (0.145)	0.381 (0.001)	0.270 (0.027)	0.275 (0.024)	—
Hearing change	0.098 (0.419)	0.103 (0.394)	0.011 (0.929)	0.244 (0.043)	0.176 (0.151)

Values are Spearman ρ (*p*-value). RT, radiotherapy; OTT, overall treatment time; Dmax, maximum point dose. Pairwise complete observations (*n* = 68–70).

**Table 9:** Firth-penalized logistic regression for post-treatment hearing change.

Predictor	Unadjusted OR (95% CI)	*p*-Value	Adjusted OR (95% CI) ^1^	*p*-Value
Age, per 10 years	1.27 (0.81–1.99)	0.288	1.28 (0.70–2.33)	0.425
Pre-treatment hearing loss	2.25 (0.75–6.72)	0.148	2.04 (0.52–8.11)	0.309
Concurrent chemoradiotherapy	1.66 (0.60–4.57)	0.331	1.66 (0.54–5.10)	0.373
Mean cochlear Dmax, per 10 Gy	1.22 (0.80–1.86)	0.349	1.31 (0.82–2.08)	0.253
Concurrent cisplatin	1.50 (0.57–3.90)	0.409	—	—
Induction chemotherapy	0.91 (0.32–2.62)	0.863	—	—
Clinical T4 category	1.70 (0.55–5.26)	0.353	—	—

OR, odds ratio; CI, confidence interval; Dmax, maximum point dose. ^1^ Adjusted model (*n* = 69) includes age, pre-treatment hearing loss, concurrent chemoradiotherapy, and mean cochlear Dmax; apparent area under the curve is 0.669 (95% CI 0.534–0.788).

**Table 10:** Post hoc parsimonious Firth-penalized logistic regression including tumor site (*n* = 69).

Predictor	Model A: Site + Dose, OR (95% CI)	*p*-Value	Model B: Site + Dose + CRT, OR (95% CI)	*p*-Value
Oropharyngeal primary (vs. other sites)	3.86 (1.27–11.7)	0.017	4.02 (1.28–12.6)	0.017
Mean cochlear Dmax, per 10 Gy	1.12 (0.69–1.82)	0.645	1.09 (0.66–1.80)	0.735
Concurrent chemoradiotherapy	—	—	1.21 (0.38–3.85)	0.748

OR, odds ratio; CI, confidence interval; CRT, concurrent chemoradiotherapy; Dmax, maximum point dose. Likelihood-ratio test for adding mean cochlear Dmax to a site-only model: *p* = 0.64. Apparent area under the curve: Model A 0.705 (95% CI 0.578–0.820); Model B 0.712 (95% CI 0.584–0.826).

**Table 11:** Sensitivity analyses for the association between concurrent chemoradiotherapy and post-treatment hearing change.

Analysis Population	*n*	CRT, *n*/*N* (%)	RT Alone, *n/N* (%)	OR (95% CI)	*p*-Value
Primary (all patients)	70	28/48 (58.3)	10/22 (45.5)	1.68 (0.61–4.49)	0.439
Curative-intent treatment only	65	28/47 (59.6)	7/18 (38.9)	2.32 (0.76–6.63)	0.170
Excluding deaths and abandonment	67	28/46 (60.9)	10/21 (47.6)	1.71 (0.61–4.68)	0.426
Excluding interrupted radiotherapy	64	27/44 (61.4)	9/20 (45.0)	1.94 (0.67–5.42)	0.281
No pre-treatment hearing loss	18	6/15 (40.0)	1/3 (33.3)	1.33 (0.12–10.89)	1.000
Excluding carboplatin-only patients	65	24/43 (55.8)	10/22 (45.5)	1.52 (0.54–4.12)	0.446

CRT, concurrent chemoradiotherapy; RT, radiotherapy; OR, odds ratio; CI, confidence interval. E-value for the primary point estimate: 1.92 (the confidence interval includes the null).

## Data Availability

The data presented in this study are available upon reasonable request from the corresponding author. The data are not publicly available due to patient privacy restrictions.
